# An Innu poem as a reflection on innovation within One Health initiative at the Université de Montréal

**DOI:** 10.1093/biosci/biaf001

**Published:** 2025-03-04

**Authors:** Olivier Beauchet

**Affiliations:** Departments of Medicine; One Health Initiative, Université de Montréal, Montréal, Québec, Canada

“And then we will say that this is where we were in that time. A place that resembles now.”

This original Joséphine Bacon's Innu text (Quebec, Canada) is a short poetic message (Bacon 2009). The Innu are a group of Indigenous people in Canada, primarily located in the province of Quebec (Clément 2018). The Innu-aimun language is central to their cultural identity and reflects their intimate relationship with the natural world. Bacon's poem invites a reflection on the interactions of human and all living forms with their habitat while incorporating the dimension of time. By examining this poem through the dual perspectives of Innu cultural values and the Western mesological approach, we have uncovered fresh insights into contemporary ecological challenges, paving the way for innovative actions within the One Health initiative of the Université de Montréal.

Innu culture emphasizes the profound interconnectedness of all living forms, including humans, animals, and plants within a geographic location (Berninger et al. 2009, Clément 2018). In Bacon's poem, the *place* is not simply a geographic location. For the Innu, it is far more than a physical entity used as material support; it is a living being actively participating in life and collective existence (Clément 2018). This perspective is rooted in a holistic worldview, where every component (i.e., humans, animals, plants, and even minerals) is interconnected. The Innu view health as relational, encompassing the well-being of the entire ecosystem. Human health, in their perspective, cannot be separated from the health of animals and the environment (Clément 2018). Through their oral traditions, the Innu preserve ecological and health-related wisdom, offering valuable insights into managing biodiversity and adapting to environmental changes, which are critical considerations for addressing modern public health and conservation challenges. For the Innu, the geographic location is not only a living territory but an entity with which they share a profound spiritual and symbolic relationship (Bacon 2009, Berninger et al. 2009, Clément 2018). Their traditions emphasize the sustainable use of natural resources, fostering ecological balance. Practices such as hunting, gathering, and land stewardship are guided by respect and ethical responsibility, minimizing harm and nurturing ecosystem resilience (McPherson 2011, Clément 2018). The moral responsibilities embedded in Innu culture enrich the ethical foundation of One Health actions, promoting equitable and respectful engagement with human communities and nature. This interconnected worldview aligns seamlessly with the principles of One Health, which recognize the deep interdependence between the health of humans, animals, plants, and all ecosystems (Faijue et al. 2024).

Moreover, the Innu hold a circular perception of time, where the past is never truly gone but remains present in everyday life (McPherson et al. 2011, Clément 2018). The line form of the poem “A place that resembles now” illustrates this perspective. Past events continue to share the present, as they are inscribed in a continuity of memory shared among human, nonhuman, and territory. This connection to time is conveyed through Innu oral traditions, which describe the territory not merely as a physical location but as a reservoir of stories, meanings, and interwoven health systems. The poem refers to this collective memory, emphasizing that the bond to the territory transcends generations, even in the face of ecological and cultural upheaval. The Innu's circular perception of time, where the past informs and remains part of the present, aligns with the One Health perspective, which highlights the interdependence of human, animal, and plant health (Faijue et al. 2024). By presenting the territory as a living repository of stories, meanings, and health systems, the poem reinforces the idea that health and well-being are deeply rooted in the cultural, ecological, and historical contexts of a place. This acknowledgment of collective memory and generational ties to the territory underscores the holistic approach essential to One Health, particularly in addressing contemporary ecological and cultural challenges.

The Western mesological approach, conceptualized by Augustin Berque, offers a complementary theoretical framework for analyzing the message in Joséphine Bacon's poem, fostering new reflections for the One Health initiative in Montreal (Berque 1996). Mesology emphasizes the concept of milieu, which is a dynamic and coconstructed interface of exchanges, meaning a back-and-forth relationship between humans and their place of living. By recognizing that ecosystems are not static backdrops but dynamic systems transformed by human practices, mesology highlights the importance of context-sensitive strategies for health and ecological management (Berque 1996, Parise et al. 2022). Furthermore, health interventions become more effective and impactful when aligned with cultural narratives and environmental perceptions, fostering greater acceptance and relevance (Vincze et al. 2021). This perspective transcends the classical dichotomy of humans versus nature, framing health and ecology as interconnected processes shaped by cultural, social, and biological exchanges (Berque 2014, Vincze et al. 2021). By emphasizing the symbolic and experiential meanings of the environment, mesology reveals how cultural narratives influence health behaviors, policies, and environmental stewardship (Berque 2014). From this viewpoint, the *place* in this poem is not merely an objective physical space but a trajective place of living, meaning a place transformed by human meanings, practices, and representations (Berque 2014). This trajective process suggests that the environment is interpreted and experienced differently across cultures, reinforcing its trajectivity and enhancing its ability to articulate meaningful relationships between the physical components of the milieu and the beings inhabiting it. In Joséphine Bacon's poem, this trajectivity is evident: The described place derives its significance through its interaction with human memory and experience.

Innu culture and mesology bridge the gap among scientific, cultural, and philosophical perspectives, fostering holistic solutions to contemporary complex ecological-related health challenges such as zoonotic diseases or climate-related health risks. Together, these approaches offer transformative value to One Health initiatives. For instance, Innu values of respect and interconnectedness enhance the moral foundations of health interventions, promoting inclusivity and equity. Similarly, a mesological lens ensures that health policies are attuned to the lived experiences and symbolic relationships of communities with their environments. Innu culture and mesology encourage systemic and creative solutions to health challenges, addressing not only biological factors but also the sociocultural and ethical dimensions of health. The intersection of Innu worldviews, mesology, and One Health brings relational ethics, ecological knowledge, and cultural sensitivity to the forefront, offering an opportunity to reimagine contemporary ecology and health systems.

Joséphine Bacon's poem serves as a poignant reminder of the deep interweaving between health and ecology. Ultimately, it reminds us that the current ecological crisis is not merely a material or health crisis but also a crisis of meaning. Addressing contemporary ecological and health challenges requires an integrated approach that blends scientific, cultural, and mesological perspectives. Understanding the environment as a coconstructed network of meanings shared with all beings inhabiting it captures the essence of One Health, promoting sustainable coexistence and well-being for all, human and nonhuman alike.

## Author Biography

Olivier Beauchet (olivier.beauchet@umontreal.ca) is affiliated with the Departments of Medicine and with the One Health Initiative at the Université de Montréal, in Montréal, Québec, Canada.

## References

[bib1] Bacon J. 2009. Bâtons à Message: Tshissinuashitakana. Mémoire D'Encrier.

[bib2] Berninger K, Kneeshaw D, Messier C. 2009. The role of cultural models in local perceptions of SFM: Differences and similarities of interest groups from three boreal regions. Journal of Environmental Management 90: 740–751. 10.1016/j.jenvman.2008.01.004 18321636

[bib3] Berque A. 1996. Être Humain sur la Terre. Gallimard.

[bib4] Berque A. 2014. Poétique de la Terre: Histoire Naturelle et Histoire Humaine. Belin.

[bib5] Clément D. 2018. Les Récits de Notre Terre: Les Innus. Presses de l'Université Laval.

[bib6] Faijue DD, Segui AO, Shringarpure K, Razavi A, Hasan N, Dar O, Manikam L. 2024 Constructing a One Health governance architecture: A systematic review and analysis of governance mechanisms for One Health. European Journal of Public Health 34: 1086–1094. 10.1093/eurpub/ckae124 PMC11631453 39214916

[bib7] McPherson DH, Douglas Rabb J. 2011. Indian from the Inside: Native American Philosophy and Culture Renewal, 2nd ed. McFarland.

[bib8] Parise AG, de Toledo GRA, Oliveira TFC, Souza GM, Castiello U, Gagliano M, Marder M. 2022. Do plants pay attention? A possible phenomenological-empirical approach. Progress in Biophysics and Molecular Biology 173: 11–23. 10.1016/j.pbiomolbio.2022.05.008 35636584

[bib9] Vincze L, Barnes K, Somerville M, Littlewood R, Atkins H, Rogany A, Williams LT. 2021. Cultural adaptation of health interventions including a nutrition component in Indigenous peoples: A systematic scoping review. International Journal for Equity in Health 20: 125. 10.1186/s12939-021-01462-x PMC8140502 34022886

